# Magnetic resonance guided focused ultrasound for noninvasive pain therapy of osteoid osteoma in children

**DOI:** 10.1186/2050-5736-3-S1-O48

**Published:** 2015-06-30

**Authors:** Adam Waspe, Yuexi Huang, Ruby Endre, Joao Amaral, Joost de Ruiter, Fiona Campbell, Charles Mougenot, Kullervo Hynynen, Gregory Czarnota, James Drake, Michael Temple

**Affiliations:** 1 Hospital for Sick Children, Toronto, Canada; 2 Sunnybrook Health Sciences Centre, Toronto, Canada; 3 Philips Healthcare, Toronto, Canada

## Background/introduction

Osteoid osteoma (OO), a small painful benign bone tumor, is the most common bone tumor in children. Pain is managed with nonsteroidal anti-inflammatory drugs but minimally invasive techniques, such as CT-guided laser ablation, have become a standard intervention. However, the potential for non-target injury is a concern as tissue temperature cannot be measured with CT and the laser induces temperatures >90°C for 10 minutes. It also includes risks from exposure to ionizing radiation, fracture, infection and transmitted thermal damage from the access needle. Magnetic resonance guided high intensity focused ultrasound (MRgHIFU) has been used successfully in small cohorts of adults with OO. The noninvasive nature of the energy means that procedures do not need to be conducted in a sterile environment since there is no mechanical penetration of the bone, reducing the chance of pathologic fracture and infection.

## Methods

A Philips Sonalleve MRgHIFU device is being used to thermally ablate OO in pediatric patients. MR provides excellent soft tissue contrast, which enhances the interface between bone and surrounding soft tissues as well as the highly vascularized core of the OO, known as the nidus. The nidus is the primary target of thermal OO treatments since destroying it will prevent regrowth of this painful lesion. Ten patients will be recruited and complete age-appropriate and validated surveys (Pediatric Ouch, and PedsQLTM) to determine how lesion/bone pain and medication usage affects health-related quality of life (HRQL) metrics, such as physical, emotional, social, and school functioning. A planning MRI will be used to ensure lesion accessibility/patient eligibility and MR thermometry will measure temperature in the target and surrounding tissue to ensure patient safety. As thermal bone ablation is painful, patients will be under general anesthesia for the treatment. Follow-up on days 2, 7, 14, 30, 90 and 180 following treatment will record pain, HRQL, and drug usage. Clinical visits on days 30, 90 and 180 will comprise a physical examination and a diagnostic MRI of the OO. Contrast enhanced MRI will indicate non-perfused tissues corresponding to the ablated tissue volume, which should be fully resolved by day 180.

## Results and conclusions

**Figure 1:**
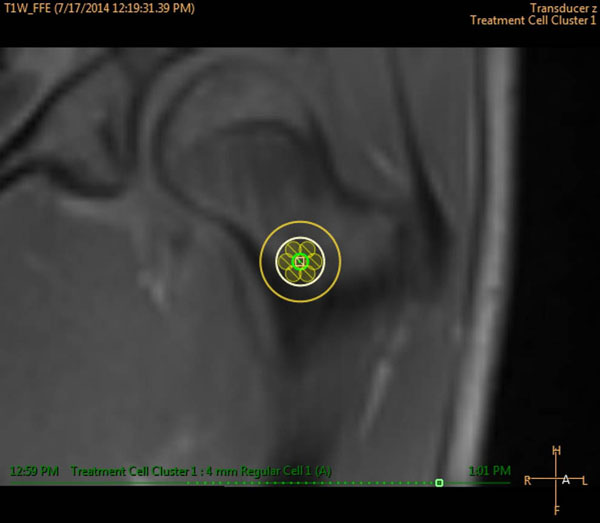
Planned treatment cells to cover the volume of the osteoid osteoma. A total of seven 4mm treatment cells, arranged in a circular cluster, covered the extent of the 1cm lesion.

**Figure 2:**
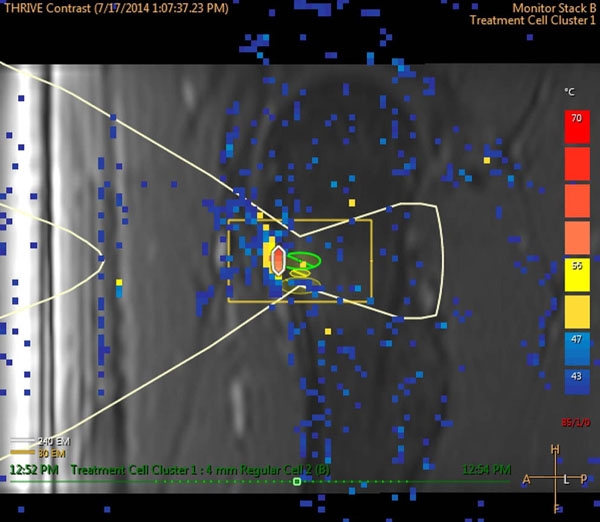
Representative thermal map from one of the treatment sonications. A 50W exposure produced a maximum temperature above 55°C at the bone surface. A small region (approximately 1 x 4 mm) adjacent to the osteoid osteoma reached sufficient temperatures to achieve a thermal dose of 240EM@43°C, causing necrosis.

**Figure 3:**
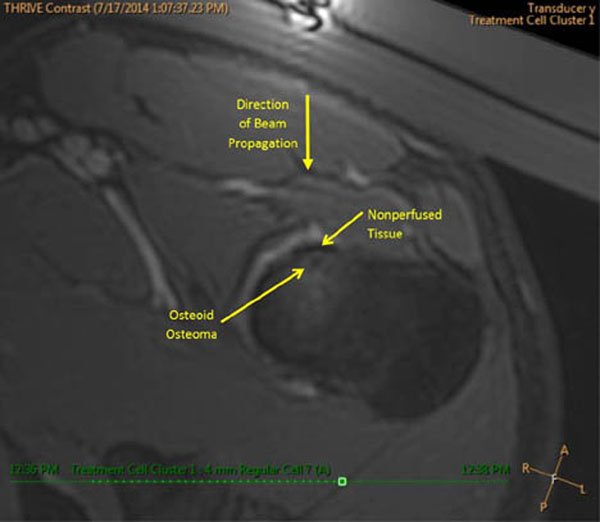
Gadolinium enhanced T1-w image of the osteoid osteoma and treatment area following thermal ablation. A small region of non-perfused tissue is visible at the bone surface, adjacent to the osteoid osteoma lesion that is approximately the same extent as the thermal dose contour.

One patient with a 1cm OO on the left femoral head is currently enrolled in the study and was treated with MRgHIFU using seven 4mm treatment cells (Fig 1). Individual treatments were 12s in duration with a power of 40-60W. Temperatures >55°C were measured at the bone surface and a thermal dose of 240EM@43°C was achieved (Fig 2). A small region of non-perfused tissue was observed in contrast enhanced MRI corresponding to the thermal dose margins (Fig 3). One week after treatment the patient is pain free, off medication, and is consistently sleeping throughout the night. Study assessment of pain, HRQL and medication will follow. It is expected that MRgHIFU will be effective for reducing pain and medication usage, leading to an improvement in HRQL.

